# Peripheral Nerve Blockade for Open Inguinal Hernia Repair in a Patient With Severe Cardiopulmonary Disease

**DOI:** 10.7759/cureus.56646

**Published:** 2024-03-21

**Authors:** Andrew S Braun, J Drake Wakefield, Promil Kukreja, Jeffrey Simmons, Beomjy Ohlman, Britney Corey, Asaf Gans

**Affiliations:** 1 Department of Anesthesiology and Perioperative Medicine, University of Alabama at Birmingham (UAB), Birmingham, USA; 2 Department of Surgery, University of Alabama at Birmingham (UAB), Birmingham, USA

**Keywords:** anatomy, lumbar plexus, local anesthesia, acute pain management, paravertebral space, paravertebral block (pvb), inguinal hernia repair, academic anesthesiology, regional anesthesiology

## Abstract

Patients with severe cardiopulmonary morbidity present a unique challenge to peri- and intraoperative providers. Inducing general anesthesia in this patient population poses the risk of adverse events that could lead to poor surgical outcomes, prolonged debilitation, or death. Therefore, it is important that anesthesiologists become comfortable with preoperative evaluation as well as alternative strategies to providing surgical anesthesia. This case report details the clinical course of a patient with severe cardiopulmonary morbidity who underwent open inguinal hernia repair without oral or intravenous sedation after receiving multi-level paravertebral blocks in addition to isolated ilioinguinal and iliohypogastric nerve blocks. This medically challenging case provides educational value regarding preoperative evaluation, pertinent anatomy and innervation, and the importance of patient-centered care and communication.

## Introduction

As obesity and severe cardiopulmonary disease become more prevalent, physicians are increasingly tasked with providing care to medically complex patients [1-3]. Specifically, anesthesiologists are more frequently challenged with providing peri- and intraoperative care to patients with poor functional status. Modern-day anesthetic care requires providers to navigate intricate pathophysiology, polypharmacy, and multimorbidity; therefore, patient safety and risk stratification have become cornerstones in the field of anesthesiology [4].

Approximately 200 million adults worldwide undergo noncardiac surgery each year; of these, an estimated 10 million sustain a major cardiac event within 30 days of surgery [5]. In the United States, the incidence of major adverse cardiac events is estimated to occur during one in 33 hospitalizations following noncardiac, inpatient surgery [6]. While several risk stratification tools exist to help guide the preoperative management of patients, it was shown in the Measurement of Exercise Tolerance before Surgery (METS) study that it is difficult to appropriately identify high-risk patients through subjective assessment [7]. The American Society of Anesthesiologists (ASA) Physical Status Classification system has been shown to be a reliable predictor of perioperative morbidity, though it was not developed with the intention of predicting patient outcomes [8]. 

The Duke Activity Status Index (DASI) is a validated risk stratification tool used at our institution in which patients self-report surrogate markers of functional status. A calculated DASI score of greater than 34 is associated with reduced odds of 30-day death or myocardial injury and one-year death or new disability [9-10]. Additionally, our institution routinely uses the Gupta Perioperative Risk for Myocardial Infarction or Cardiac Arrest (MICA). MICA provides a percentage risk of myocardial infarction or cardiac arrest intraoperatively or up to 30 days postoperatively based on preoperative risk factors [11]. Likewise, the ARISCAT (Assess Respiratory Risk in Surgical Patients in Catalonia) score for postoperative pulmonary complications is used at our institution. This provides a numerical score based on preoperative metrics where a score of less than 26 corresponds to low risk of postoperative pulmonary complications, a score of 26-45 corresponds to intermediate risk, and a score of greater than 45 corresponds to high risk [12].

This case report describes a surgical patient who presented to our institution for an open inguinal hernia repair. His severe cardiopulmonary disease and debilitation made him a very challenging surgical patient for anesthesia providers, and extreme caution was used when planning his operative care. The purpose of this case report is to detail the patient's clinical course, explain the preoperative decision-making that was done with the patient, review pertinent anatomy and innervation, and provide feedback from the patient’s overall surgical experience. 

## Case presentation

**Figure 1:**
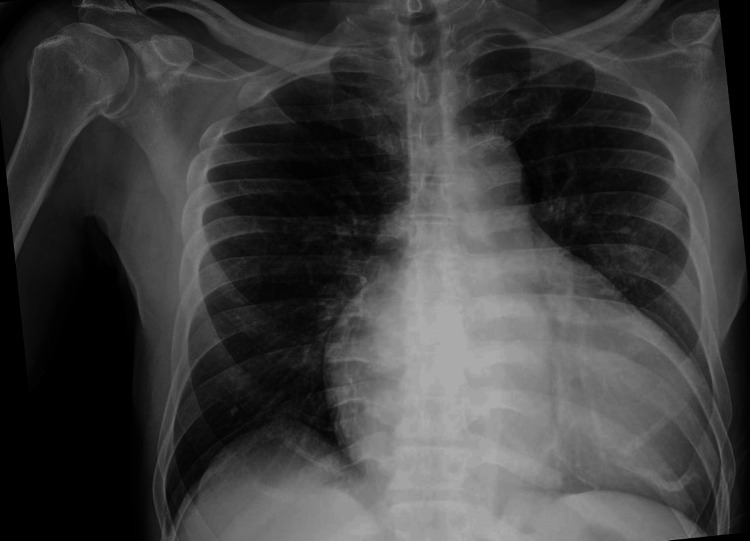
The patient's preoperative chest x-ray, which shows an enlarged cardiomediastinum.

A 64-year-old male (70 kg, 175 cm, BMI 23 kg/m2) presented to our institution for an open repair of a left inguinal hernia, which he stated was negatively impacting his quality of life. The patient suffered from several comorbid conditions such as hypertension, congestive heart failure with reduced biventricular function, atrial fibrillation, chronic obstructive pulmonary disease (COPD), pulmonary hypertension with known lung blebs, peripheral vascular disease, and an abdominal aortic aneurysm (4.6 x 5.0 cm, unrepaired, under serial surveillance). The patient was prescribed apixaban for stroke prevention due to his history of atrial fibrillation, which he was compliant with. Additionally, the patient had poorly controlled type 2 diabetes mellitus and a longstanding history of tobacco use. Cardiology consultation prior to surgery recommended implantable cardioverter defibrillator (ICD) placement, which the patient declined. The patient was known to have a difficult airway from prior tracheal intubation attempts at our institution. At his preoperative clinic visit, the patient’s DASI was calculated to be 13.45, MICA 2.5%, and ARISCAT score 11 points. Figure 1 shows the patient's preoperative chest x-ray, depicting cardiomegaly and an enlarged mediastinum. 

This patient’s preoperative transthoracic echocardiogram (TTE) showed severely reduced left ventricular (LV) function (LV ejection fraction <20%), a small LV thrombus, grade III diastolic dysfunction, moderate mitral valve regurgitation, elevated right ventricular systolic pressures (40 mmHg), severe tricuspid regurgitation, and mild pulmonary artery dilation. Video 1 shows the patient's TTE in the parasternal long-axis view, which depicts a poorly functioning LV as well as a thickened left ventricle and interventricular septum. 

**Video 1:** The patient's preoperative TTE in the parasternal long-axis view, showing the left atrium, left ventricle, left ventricular outflow tract, and right ventricle. TTE: Transthoracic echocardiogram.

Video 2 shows the patient's TTE in the apical four-chamber view. This video shows the poor contractility of both the right and left ventricles, as well as bilateral atrial enlargement. 

**Video 2:** The patient's preoperative TTE in the apical four-chamber view, showing the right and left atria and the right and left ventricles. TTE: Transthoracic echocardiogram.

Video 3 shows the patient's TTE in parasternal short-axis view with emphasis on the LV, again showing the poor contractile force of the left ventricle.

**Video 3:** The patient's preoperative TTE in parasternal short-axis view, showing the left ventricle in cross-section. TTE: Transthoracic echocardiogram.

Upon preoperative evaluation, the patient endorsed shortness of breath with exertion, though he denied using supplemental oxygen at home. Due to the patient’s disease state and the likelihood of adverse cardiopulmonary events under general anesthesia (GA), a thorough discussion was had with the patient regarding the risks and benefits of different modalities of anesthesia including general endotracheal anesthesia, monitored anesthesia care (MAC) with peripheral nerve blockade (PNB), and neuraxial anesthesia (NA). Ultimately, the patient and anesthesia provider agreed to proceed with PNB with the intention of creating a surgical block for his procedure.

**Figure 2:**
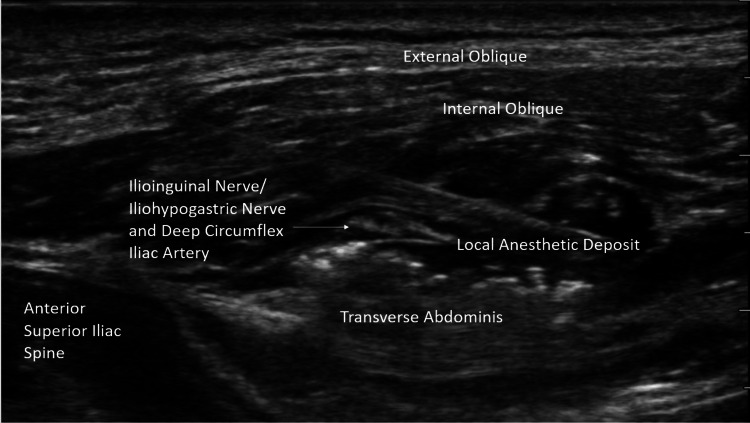
An ultrasound image of the patient's ilioinguinal/iliohypogastric nerve block was captured at the time of the procedure.

After ensuring appropriate discontinuation of apixaban according to the American Society of Regional Anesthesia and Pain Medicine (ASRA) guidelines (72 hours) [13], this patient underwent left-sided T11, T12, and L1 landmark-based paravertebral nerve blocks (PVB) in the sitting position. Given the concern for worsening pulmonary hypertension, this was done without the utilization of intravenous or oral sedation. An amount of 5 mL of 0.5% ropivacaine without additives was injected into the paravertebral space at each level (15 mL total) utilizing nerve stimulation to ensure appropriate dermatomal coverage and proximity to the nerve root. Cautionary measures, such as aspiration prior to injection, were taken throughout to avoid intravascular or pleural injection. In addition to PVB, the patient underwent an isolated left ilioinguinal/iliohypogastric nerve block with 10 mL of 0.5% ropivacaine without additives (Figure 2). 

The nerve block was tested using ice after 20 minutes, and the patient was found to be insensate on the surgical side in the pattern of the T10-L2 dermatomes without evidence of quadriceps weakness. Once in the operating room, the patient successfully underwent open repair of his left inguinal hernia without any sedation. The intraoperative care from the anesthesia team included supplemental oxygen (with end-tidal CO2 monitoring), administration of antibiotics prior to the procedure, and monitoring of vital signs in accordance with ASA standards for MAC. The estimated blood loss for this surgical procedure was 10 mL. No supplemental local anesthetic was utilized by the surgical team. The surgical duration was 75 minutes. The patient suffered no postoperative complications in the post-anesthesia care unit (PACU) and was discharged home on the day of surgery. 

Approximately three weeks following surgery, members of the anesthesia team contacted the patient, and consent was granted to write this case report. At the time of follow-up, the patient had not experienced any adverse events such as new or worsening heart failure symptoms, increased shortness of breath, surgical complications, or hospital re-admission. The patient stated his overall experience was good, and he was grateful to the peri- and intraoperative providers whom he trusted with his care.

## Discussion

This medically challenging case highlights the importance of developing advanced skills for both preoperative evaluation and intraoperative management of patients. This patient's poor functional status was characterized by both his symptom burden and preoperative risk assessment tools (MICA and DASI score). Supplemental preoperative workup, including his chest x-ray, preoperative electrocardiogram, and recent echocardiogram were helpful for developing an anesthetic plan. Additionally, he had been seen in our institution's cardiology transitional clinic prior to surgery, which addressed the importance of pre-habilitation via increasing physical exercise as tolerated. No further counseling on preoperative nutrition or psychological preparation for surgery is noted. 

**Figure 3:**
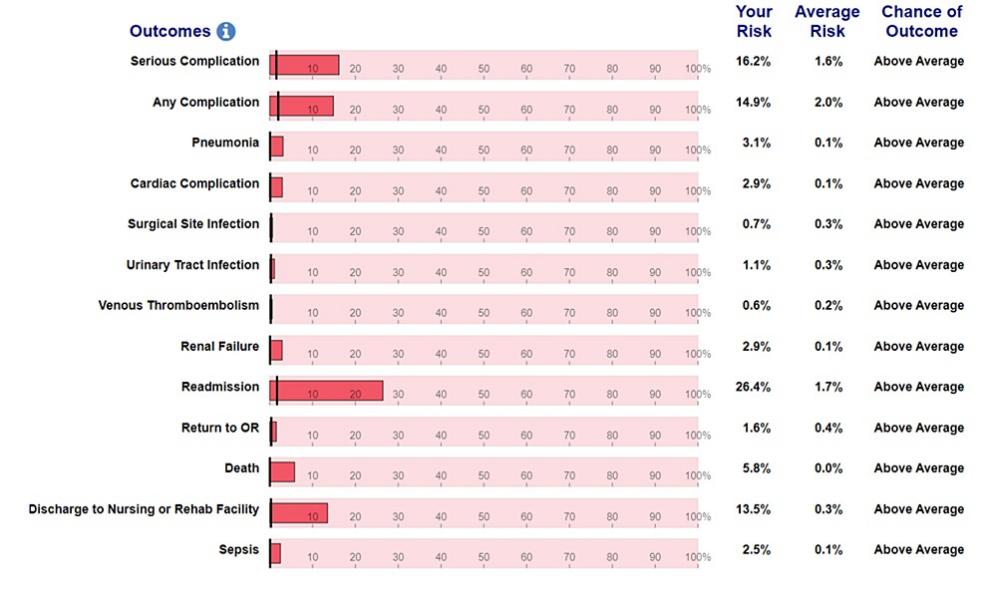
American College of Surgeons Surgical Risk Calculator displaying a comprehensive list of possible postoperative complications for this patient.

While severe cardiopulmonary disease is not an absolute contraindication to GA, inducing anesthesia in this patient posed several critical risks, which were discussed with the patient. His cardiac history put him at risk for myocardial ischemia, arrhythmia, and cardiac arrest while under anesthesia. His pulmonary history put him at risk for hypoxic events, right heart strain, and prolonged intubation requiring intensive care. Aside from cardiopulmonary complications, the American College of Surgeons Surgical Risk Calculator categorized this patient as an “above average” risk for postoperative sepsis, acute renal failure, pneumonia, and hospital readmission (Figure 3) [14]. 

Because of these risks, alternative options, such as NA and PNB, were also discussed with the patient. While neuraxial techniques have long been used for noncardiac surgery, NA has failed to demonstrate a significant reduction in 30-day mortality, adverse cardiac events, myocardial infarction, incidence of pneumonia, blood transfusion requirement, or length of stay when compared to GA [15]. For this patient specifically, the risk of high spinal, and hemodynamic compromise from sympathectomy, and bleeding complications deterred the patient and provider from favoring NA for this surgery. After continued discussion, surgical blockade via PNB was thought to be the safest option for the patient. Table 1 includes a summary of many of the risks and benefits of anesthesia care that were considered by the patient before proceeding with his surgery.

**Table 1:** Risks and benefits of general anesthesia, neuraxial anesthesia, and regional anesthesia as discussed with the patient prior to proceeding to the operating room.

Technique	Benefits	Risks
General anesthesia	Effective, akinesis, amnestic effect, secured airway	Known difficult airway, hemodynamic compromise, positive pressure ventilation
Neuraxial anesthesia	Effective, no need for airway instrumentation	Hemodynamic compromise, possible hematoma formation, potential need to convert to general anesthesia
Regional anesthesia	Prolonged analgesia, no hemodynamic compromise, no need for airway instrumentation	Unreliable local anesthesia spread, potential need to convert to general anesthesia, injury from needle placement

Of note, the anesthesia provider for the case discussed a secondary plan if PNB were to fail, including aborting the surgical procedure altogether vs. performing an inhalational induction of GA followed by laryngeal mask airway placement with maintenance of spontaneous ventilation. The patient was counseled on the need for a pre-induction arterial line in the case he would require GA.

There are several reasons why PVB was the regional technique chosen for this patient. The paravertebral space, which is located directly adjacent to the neural foramen of the spine, houses spinal nerve roots as they divide into anterior and ventral rami [16]. Performing nerve blockade within this space provides a fast and dense sensory deficit due to its lack of fascial covering [16]. Because of this, PVB has been used as surgical anesthesia for awake surgery for a variety of operations including breast and hernia surgery [16,17]. Furthermore, PVB has been shown to reduce postoperative pain, reduce nausea and vomiting, and improve patient satisfaction when compared to GA [17]. PVB has been credited as achieving analgesia comparable and non-inferior to epidural analgesia and is known to have a low block failure and complication rate (2.8% and 1.2%, respectively) [18,19]. 

Three adjacent low-volume injections were performed at the T11, T12, and L1 paravertebral spaces with the expectation that local anesthetic would spread to one additional level bidirectionally, thus anesthetizing the T10-L2 dermatomes. This is consistent with what the patient experienced. The ilioinguinal and iliohypogastric nerves were blocked separately in order to anesthetize the skin of the proximal, medial thigh, and anterior scrotum [20]. Together, these techniques allowed the surgery to be performed without any intravenous sedation or analgesia. It is noteworthy that in the instance of successful PVB, the ilioinguinal and iliohypogastric nerves did not need to be blocked separately; however, due to the risk of block failure associated with all regional anesthetic techniques, these nerves were blocked to ensure the patient would not require any supplemental analgesia or sedation. 

Quadratus lumborum (QL) block has been used as the primary anesthetic for open hernia repair in past case reports due to its broad dermatomal coverage as well as visceral analgesic benefit [21,22]. QL block, however, is associated with a higher block failure rate compared to PVB and thus was not the regional approach chosen for this patient [23].

When attempting to achieve a surgical block using regional anesthesia techniques, assessing the spread and density of the block, as well as frequent discussion with the patient, is crucial to providing excellent patient-centered care. In this case, an assessment of the nerve block was performed using an ice test prior to the procedure starting. Our institution routinely uses ropivacaine for regional anesthesia due to its long duration of action and lower lipophilicity compared to bupivacaine, thus lowering the likelihood of cardiac or central nervous system toxicity [24]. When providing surgical blockade, it is important to always calculate weight-based toxic doses given that high concentrations of local anesthetic may be used. The patient described in this case report is 70 kg, and therefore the maximum dose of ropivacaine he could receive is 210 mg (assuming a toxic dose of 3 mg/kg). For his nerve blocks, he received 125 mg total of 0.5% ropivacaine (25 mL total) and therefore was under the toxic dose limit. By using less than the toxic dose, it was possible for the patient to receive additional infiltrative local anesthesia intraoperatively if needed. However, additional local anesthetic was not required. 

## Conclusions

Severely debilitated patients pose unique challenges to anesthesia providers and necessitate careful and comprehensive preoperative evaluation. Alternatives to general anesthesia, such as neuraxial and regional techniques, should be considered for these patients as these techniques may improve outcomes. As the field of anesthesia evolves, providers must develop and maintain these skills to enable the proper care of challenging patients. Finally, this challenging case emphasizes the importance of tailoring anesthetic plans to patient needs as well as providing patients with thorough discussion, education, and expectations. 
